# Trends in Real Estate Investment Trust Ownership of US Health Care Properties

**DOI:** 10.1001/jamahealthforum.2022.1012

**Published:** 2022-05-13

**Authors:** Joseph Dov Bruch, Tamar Katz, Tarun Ramesh, Eileen Appelbaum, Rosemary Batt, Thomas C. Tsai

**Affiliations:** 1 Department of Health Care Policy, Harvard Medical School, Boston, Massachusetts; 2 Center for Economic and Policy Research, Washington, DC; 3 Harvard Medical School, Boston, Massachusetts; 4 ILR School, Cornell University, Ithaca, New York; 5 Department of Health Policy and Management, Harvard T.H. Chan School of Public Health, Boston, Massachusetts; 6 Department of Surgery, Brigham and Women's Hospital, Boston, Massachusetts

## Abstract

This cross-sectional study examines the prevalence and characteristics of real estate investment trust–owned health care properties in the US health care sector.

## Introduction

Against a backdrop of continued consolidation in US health services, real estate investment trusts (REITs) have emerged as an understudied force facilitating the financialization of the American health care system. Real estate investment trusts are corporate entities that invest in real estate assets. Like mutual funds, REITs facilitate collective investment and operate as pass-through vehicles for the benefit of investors.^1^ In 2021, REITs owned more than $3.5 trillion in US assets, ranging from residential and retail real estate to specialty sectors like health care.^2^

Health care–focused REITs own a portfolio of income-producing real estate and generate profit by acquiring properties (eg, hospitals) and leasing the real estate back to the health care facility tenant (ie, a sale-leaseback). This transaction generally occurs through a 10-year, triple net lease in which the tenant is responsible for facility rent, maintenance, insurance, and taxes.^3^

To our knowledge, there is no research on the association of REIT acquisitions with health care delivery. Moreover, it is unknown if REITs are a minor or growing factor in the health care system. This study evaluates the prevalence of REIT-owned health care properties in the US health care sector and describes characteristics of REIT-owned hospitals.

## Methods

We identified the number of health care properties in 2021 owned by REITs (eMethods 1 in the Supplement). We then located hospitals owned by REITs and identified the year during which their real estate was acquired. A multivariate logistic regression was then used to assess which hospital characteristics had the largest association with REIT ownership (eMethods 2 in the Supplement). Because information on most of the REIT-owned properties was publicly available, the data likely provide an accurate, aggregate count of REIT-owned properties, but the total properties within each subsector were estimates across various sources (eMethods 3 in the Supplement). This study did not include human participants and was not subject to an institutional review board. The study followed the Strengthening the Reporting of Observational Studies in Epidemiology (STROBE) reporting guideline for cross-sectional studies. Statistical analysis was conducted using R, version 4.1.0 (R Foundation).

## Results

In 2021, REITs owned 197 (3%) of all hospitals and 1870 (12%) of all skilled nursing facilities (Table). Real estate investment trust hospital acquisitions have increased during the past 15 years until the COVID-19 pandemic, during which acquisitions were minimal (Figure). In a multivariate logistic analysis, some of the characteristics most strongly associated with REIT ownership were for-profit status (odds ratio, 25.05; 95% CI, 14.69-42.70; *P* < .001) and urban status (odds ratio, 6.8; 95% CI, 1.61-28.67; *P* = .01).

**Table.** REIT-Owned Health Care Properties in the US

Hospital characteristics	REIT-owned hospitals, No. (%)		OR (95% CI)	*P* value^a^
	Yes	No		
Hospitals^b^	152^b^	5683		
Patients, %				
Medicare	53	48	2.42 (0.50-11.76)	.27
Medicaid	15	18	0.70 (0.08-5.99)	.75
For profit	133 (88)	1410 (25)	25.05 (14.69-42.70)	<.001
Teaching status	33 (22)	1819 (32)	0.92 (0.58-1.46)	.73
Urban	150 (99)	4606 (81)	6.8 (1.61-28.67)	.01
Hospital type				
Acute care				
General	86 (57)	4246 (75)	1 [Reference]	
Long-term	23 (15)	328 (6)	0.56 (0.31-1.00)	.05
Rehabilitation	28 (18)	276 (5)	0.83 (0.49-1.42)	.50
Other	15 (10)	833 (15)	0.32 (0.16-0.62)	<.001
Region				
Northeast	25 (16)	710 (13)	1 [Reference]	
Midwest	18 (12)	1605 (28)	0.33 (0.17-0.63)	<.001
South	58 (38)	2291 (40)	0.3 (0.18-0.50)	<.001
West	51 (34)	1077 (19)	0.84 (0.49-1.43)	.52
Size^c^				
Small	90 (59)	3223 (57)	1 [Reference]	
Medium	58 (38)	1960 (35)	1.09 (0.68-1.74)	.71
Large	4 (3)	500 (9)	0.62 (0.20-1.93)	.41
**Property type**	**Properties**			
	**REIT-owned**	**Total (% REIT-owned)^d^**		
Hospitals	197^c^	5835 (3)		
Senior housing/assisted living facilities	2619	28 900 (9)		
Medical office buildings	2515	41 000 (6)		
Skilled nursing facilities	1870	15 327 (12)		
Total	7201	91 062 (8)		

Abbreviations: OR, odds ratio: REIT, real estate investment trust.

^a^ A multivariate logistic regression was used to assess which hospital characteristics had the largest association with REIT ownership.

^b^ Data were available for 152 unique hospital provider numbers (as defined by the US Centers for Medicaid & Medicare Services) of 197 total hospitals; several hospitals within a health system filed under a single provider number or did not have data available in the American Hospital Association 2021 survey.

^c^ A total of 1 to 99 beds was considered small, 100 to 399 medium, and 400 or more large. We defined REIT-owned hospitals as those in which the REIT owned the entire real estate to a hospital or the primary hospital in a health system.

^d^ The total properties within each subsector were estimates across various sources. See eMethods 3 in the Supplement for an explanation of how percentiles were calculated.

**Figure.**
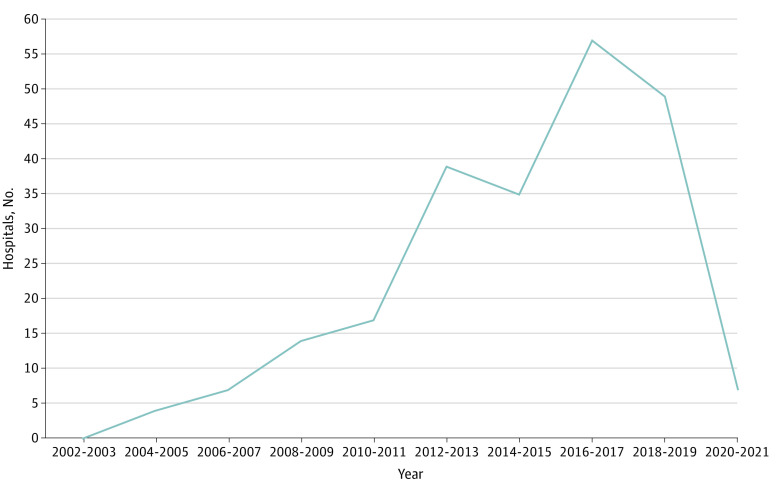
Hospitals Acquired by Real Estate Investment Trusts (REITs) From 2002 to 2021 After an REIT acquires a hospital, it will lease the real estate to the current hospital operator (sale-leaseback) or to a new hospital operator. There were 229 acquisitions across the time frame. This number differs from the 197 hospitals in the Table, which is the count of all REIT-owned hospitals in 2021. A total of 32 hospitals lost REIT ownership or closed. Each data point represents the sum of hospitals acquired by REITs across the 2-year span.

## Discussion

Real estate investment trusts have substantial ownership of US health care real estate (8%) overall, with 3% of hospitals in the US owned by REITs. Urban and for-profit hospitals were most likely to be owned by REITs. Major REIT acquisitions occur regularly; thus, our data and estimates are limited to 2021.

There is concern that REIT ownership of health care facilities may divert capital away from investments in clinical care delivery toward generating high returns for investors instead. The immediate capital gained from a sale-leaseback could theoretically be used for facility investments. However, the capital is often diverted to a holding company. Following an acquisition of a hospital, some private equity firms have sold the physical property of the hospital and its ancillary real estate to an REIT as a way of monetizing their holdings.^4^ Income from the sale-leaseback flows as dividends to the private equity firm shareholders, generating immediate investor returns but leaving the hospital with burgeoning rental fees.^5^ Pressured by mounting financial obligations, some health care operators with REIT-owned real estate may choose to close, as observed in 2018 when one of the largest nursing home chains in the US closed after selling its real estate to an REIT.^6^

To our knowledge, there is no research quantifying the association of REITs with quality of care, costs to patients, and the financial security of health care operators. Policy makers and health care operators should ensure that the REIT business model is compatible with long-term priorities in health care delivery.

## Notes

Supplement. **eMethods 1.** Identifying and Locating REIT-Owned Health Care Properties **eMethods 2.** Testing for Differences between REIT-Owned and Non-REIT-Owned Properties **eMethods 3.** Total Property Estimates Across Sectors
