# Supplementary material for: Trends in Real Estate Investment Trust Ownership of US Health Care Properties
Source: JAMA Health Forum. 2022 May 13;3(5):e221012. doi: 10.1001/jamahealthforum.2022.1012 (PMC9107031; doi:10.1001/jamahealthforum.2022.1012)
Supplement: Supplement. — eMethods 1. Identifying and Locating REIT-Owned Health Care Properties eMethods 2. Testing for Differences between REIT-Owned and Non-REIT-Owned Properties eMethods 3. Total Property Estimates Across Sectors [file jamahealthforum-e221012-s001.pdf]

Supplemental Online Content

Bruch JD, Katz T, Ramesh T, Appelbaum E, Batt R, Tsai TC. Trends in real estate investment trust ownership of US health care properties. *JAMA Health Forum*. 2022;3(5):e221012. doi:10.1001/jamahealthforum.2022.1012

eMethods 1. Identifying and Locating REIT-Owned Health Care Properties
eMethods 2. Testing for Differences between REIT-Owned and Non-REIT-Owned Properties
eMethods 3. Total Property Estimates Across Sectors

This supplemental material has been provided by the authors to give readers additional information about their work.

© 2022 Bruch JD et al. *JAMA Health Forum.*

eMethods 1. Identifying and Locating REIT-Owned Health Care Properties

According to the National Association of Real Estate Investment Trusts (NAREIT) there are only 20 health care-focused REITs in the United States, which are listed on their website. Across all our searches, we could find no other non-health care-focused REIT that owned properties across the property types we evaluated. Unlike private companies owned by private equity and venture capital, most REITS are publicly traded and thus data on facility holdings is publicly available. Using U.S. Securities and Exchange Commission documents, annual company reports, company statements, and online news reports, we collected facility information and data on all property types in 2021 owned by these REITs. Using these same sources, we found the year of acquisition for hospitals owned by REITs in 2021. For hospitals where the year of acquisition was not available online, we looked across annual reports to identify the year the hospital was introduced to the list of portfolio properties. We also included acquisitions of hospitals that were not REIT-owned in 2021, but which were previously owned by a REIT and documented within these sources.

© 2022 Bruch JD et al. *JAMA Health Forum.*

eMethods 2. Testing for Differences between REIT-Owned and Non-REIT-Owned Properties

Using the American Hospital Association 2021 survey, we performed a multivariate logistic regression was used to assess the characteristics of hospitals with the greatest association with REIT ownership, conditional on the other covariates.

p= P(Hospital is REIT-Owned)

Logit(*p*) = β_0 + β_1(For-Profit)+ β_2(Teaching Status)+ β_3(Urban Status) + β_4(% Medicare Patients)+ β_5(% Medicaid Patients) + **β(Hospital Type) + β(Region) + β(Size)**

Hospital Type included “General Acute Care,” “Long-term Acute Care,” “Rehabilitation,” and “Other.” Region included “Northeast,” “Midwest,” “South,” and “West.” Size include “Small,” “Medium,” and “Large” where 1-99 beds is “Small,” 100-399 is “Medium,” and 400+ is “Large.”

© 2022 Bruch JD et al. *JAMA Health Forum.*

eMethods 3. Total Property Estimates Across Sectors

To get estimates of the number of properties across health care subsectors in the U.S., we looked to the following sources.

1) According to the American Hospital Association 2021 Survey, there are 5,835 hospitals in the United States.[1]
2) According to the American Health Care Association and its National Center for Assisted Living, there are 28,900 assisted living facilities.[2]
3) According to a 2018 report by the CBRE Group, there are approximately 41,000 medical office buildings.[3]
4) According to the Kaiser Family Foundation, there are 15,327 skilled nursing facilities.[4]

[1] American Hospital Association. AHA Annual Survey Database™ [online] Available at: < https://www.ahadata.com/aha-annual-survey-database > [Accessed 26 October 2021].
[2] AHCA NCAL. 2021. *Facts & Figures*. [online] Available at: <https://www.ahcancal.org/Assisted-Living/Facts-and-Figures/Pages/default.aspx#:~:text=%E2%80%8BT%E2%80%8Bhere%20are,community%20is%2033%20licensed%20beds> [Accessed 26 October 2021].
[3] CBREUS. 2021. *CBRE: Strong Population Growth Leads DFW to Highest Medical Office Space Completions in U.S.*. [online] Available at: <https://www.cbre.us/people-and-offices/corporate-offices/dallas-ft-worth/dallas-fort-worth-media-center/strong-population-growth-leads-dfw-to-highest-medical-office-space-completions-in-us> [Accessed 26 October 2021].
[4] KFF. 2021. *Total Number of Certified Nursing Facilities*. [online] Available at: <https://www.kff.org/other/state-indicator/number-of-nursing-facilities/?currentTimeframe=0&sortModel=%7B%22colId%22:%22Location%22,%22sort%22:%22asc%22%7D> [Accessed 26 October 2021].

© 2022 Bruch JD et al. *JAMA Health Forum.*
